# Numerical Distributions of Parasite Densities During Asymptomatic Malaria

**DOI:** 10.1093/infdis/jiv596

**Published:** 2015-12-17

**Authors:** Mallika Imwong, Kasia Stepniewska, Rupam Tripura, Thomas J. Peto, Khin Maung Lwin, Benchawan Vihokhern, Klanarong Wongsaen, Lorenz von Seidlein, Mehul Dhorda, Georges Snounou, Lilly Keereecharoen, Pratap Singhasivanon, Pasathorn Sirithiranont, Jem Chalk, Chea Nguon, Nicholas P. J. Day, Francois Nosten, Arjen Dondorp, Nicholas J. White

**Affiliations:** 1 Mahidol Oxford Research Unit; 2 Department of Molecular Tropical Medicine and Genetics; 3 WorldWide Antimalarial Resistance Network (WWARN); 4 Department of Tropical Hygiene, Faculty of Tropical Medicine, Mahidol University, Bangkok; 5 Shoklo Malaria Research Unit, Faculty of Tropical Medicine, Mahidol University, Tak, Thailand; 6 Centre for Tropical Medicine and Global Health, Nuffield Department of Medicine, University of Oxford; 7 WWARN, Churchill Hospital, Oxford, United Kingdom; 8 Sorbonne Universités, UPMC Univ Paris 06, UPMC UMRS CR7; 9 Centre d'Immunologie et de Maladies Infectieuses–Paris, Institut National de la Santé et de la Recherche Medicale U1135–Centre National de la Recherche Scientifique ERL 8255, Paris, France; 10 National Center for Parasitology, Entomology, and Malaria Control, Phnom Penh, Cambodia

**Keywords:** malaria, *Plasmodium falciparum*, *Plasmodium vivax*, asymptomatic parasitemia, PCR

## Abstract

***Background.*** Asymptomatic parasitemia is common even in areas of low seasonal malaria transmission, but the true proportion of the population infected has not been estimated previously because of the limited sensitivity of available detection methods.

***Methods.*** Cross-sectional malaria surveys were conducted in areas of low seasonal transmission along the border between eastern Myanmar and northwestern Thailand and in western Cambodia. DNA was quantitated by an ultrasensitive polymerase chain reaction (uPCR) assay (limit of accurate detection, 22 parasites/mL) to characterize parasite density distributions for *Plasmodium falciparum* and *Plasmodium vivax*, and the proportions of undetected infections were imputed.

***Results.*** The prevalence of asymptomatic malaria as determined by uPCR was 27.5% (1303 of 4740 people tested). Both *P. vivax* and *P. falciparum* density distributions were unimodal and log normal, with modal values well within the quantifiable range. The estimated proportions of all parasitemic individuals identified by uPCR were >70% among individuals infected with *P. falciparum* and >85% among those infected with *P. vivax.* Overall, 83% of infections were predicted to be *P. vivax* infections, 13% were predicted to be *P. falciparum* infections, and 4% were predicted to be mixed infections. Geometric mean parasite densities were similar; 5601 *P. vivax* parasites/mL and 5158 *P. falciparum* parasites/mL.

***Conclusions.*** This uPCR method identified most infected individuals in malaria-endemic areas. Malaria parasitemia persists in humans at levels that optimize the probability of generating transmissible gametocyte densities without causing illness.

Asymptomatic malaria parasitemia is very common in areas of high stable transmission—indeed, everyone there may be infected. In contrast, asymptomatic parasitemia was considered relatively uncommon in areas of low seasonal transmission, which predominate in much of Asia and the Americas, but recent epidemiological studies using sensitive methods of parasite detection have revised this view [1–8]. In Myanmar, Thailand, Cambodia, Laos, and Vietnam, a substantial proportion of the population living in malaria-endemic areas harbor asymptomatic parasitemias [9–11]. These infected individuals sustain malaria over the dry season, and so they are an important source of malaria transmission and a major obstacle to elimination. However, because of the limited sensitivity of detection methods, the proportion of people in any area with chronic low-density parasitemia has been unknown. Accurate characterization of malaria epidemiology and its geographic distribution is essential in planning regional control and elimination strategies. We used a quantitative ultrasensitive polymerase chain reaction (uPCR) method of parasite quantitation in blood volumes of >200 µL [12] to characterize the numerical distributions of parasite densities in asymptomatic *Plasmodium falciparum* and *Plasmodium vivax* infections in populations living in malaria-endemic regions of western Cambodia and the Thailand-Myanmar border [11]. This method was sufficiently sensitive to identify the majority of infected persons and therefore allowed prediction of the proportion of the population with parasitemia levels below the limits of detection by the most sensitive current techniques.

## METHODS

These studies took place in malaria-endemic regions along the border between eastern Myanmar and northwestern Thailand and in western Cambodia as a prelude to the assessment of elimination interventions. In these areas, malaria transmission is low and seasonal, with entomological inoculation rates of usually <3 person/year and often <1 person/year. Most clinical cases of malaria occur during the rainy season, between May and December [1, 13–18]. In the past, *P. vivax* and *P. falciparum* each caused approximately half of the clinical cases, although with recent reductions in malaria incidence, *P. vivax* now predominates.

Full details of these epidemiological studies have been reported recently [11]. In brief, screening surveys were conducted in 73 villages, followed by detailed cross-sectional malariometric surveys in 4 selected villages located within 10 km of the Thailand-Myanmar border that were considered representative of the area in terms of environment, ecology, population and behavior. The 3 villages in the Pailin region of western Cambodia were selected because they had the highest incidence of clinical falciparum malaria in the village malaria workers' records from 2012.

### Procedures

All individuals aged ≥6 months were invited to participate in the surveys. Individual informed consent was obtained from adults, and parental consent was obtained for children aged <16 years. Demographic data and symptom information were collected from each person, and the tympanic temperature, weight, and height were measured. A venous blood specimen (volume, 3 mL) was collected from all individuals aged ≥5 years, and 500-µL blood specimens were obtained from children aged ≥6 months to 5 years.

Sample processing for quantitative uPCR analysis included separation of plasma, buffy coat, and packed red blood cells, which were then frozen and stored at −80°C and transported to the laboratory in Bangkok, Thailand, for DNA extraction and quantitative uPCR analysis [11, 12]. All samples obtained for molecular analyses were handled and processed according to the standard operating procedures developed specifically for these studies [12].

### Quantification of Malaria Parasitemia

Description, evaluation, validation, and performance characteristics of the high-volume quantitative uPCR analyses have been reported recently in detail [12]. In brief, the DNA template for *Plasmodium*-specific PCR detection and quantification was purified from thawed packed red blood cells. DNA extraction from a carefully measured volume was performed using a QIAamp Blood Mini Kit (Qiagen, Hilden, Germany) for sample volumes of packed red blood cells ≤200 µL, or a QIAamp Blood Midi Kit for sample volumes between 200 and 2000 µL. The purified DNA was dried completely in a centrifugal vacuum concentrator and then resuspended in PCR-grade water. The concentration factor was defined by the original blood volumes (100–2000 µL) divided by the double-distilled water volumes (10–50 µL). Two microliters of resuspended DNA was used as a template in the quantitative uPCR reaction. The number of parasite genomes in each sample was estimated by an absolute quantitative real-time PCR method, using the Quanti-Tect Multiplex PCR No Rox system (Qiagen). The 18S ribosomal RNA (rRNA)–targeting primers and hydrolysis probes are highly specific for *Plasmodium* species [12]. The lower limit of accurate quantitation was 22 parasites/mL of whole blood [12]. Quantification of high parasite densities (>30 000 000 parasites/mL) by means of this technique becomes progressively inaccurate.

**Figure 1.**
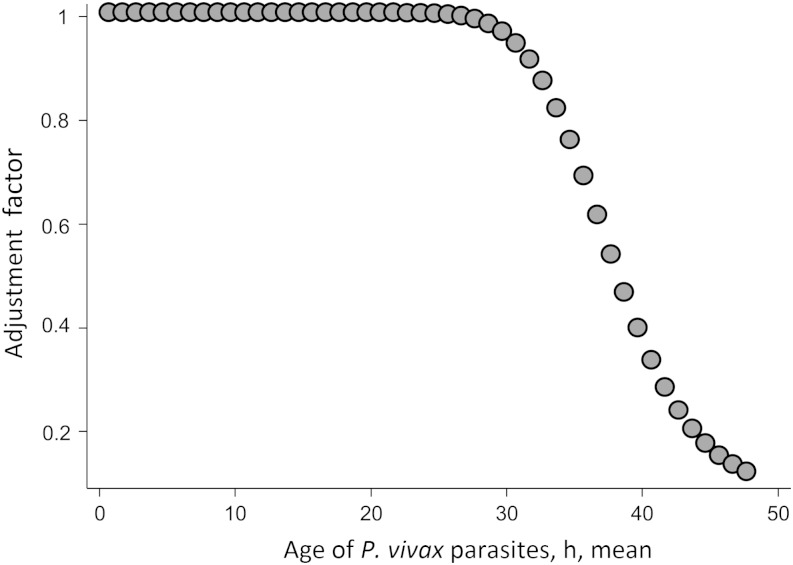
Erythrocytes containing dividing forms of *Plasmodium vivax* circulate freely, in contrast to *Plasmodium falciparum*, in which erythrocytes containing dividing forms are sequestered. This correction factor was applied to convert densities of peripheral blood *P. vivax* genomes to densities of parasites in relation to parasite age.

In uncomplicated falciparum malaria, later asexual parasite stages cytoadhere and so are seldom seen in the peripheral circulation, whereas all blood stages circulate in *P. vivax* infections. The *P. falciparum* density (asexual plus sexual stages) is approximately equivalent to the genome density, as nuclear division occurs in sequestered not circulating parasites, whereas for *P. vivax* (which does not sequester significantly), there is 1 genome/parasite for approximately 36 hours of the 48-hour cycle, followed by genome doubling every 3 hours (Figure 1). Thus, in a completely asynchronous *P. vivax* infection, there would be an approximate average of 2.63 genomes per parasite. Both asynchronous and synchronous infections were evaluated.

### Species Identification

For uPCR-positive blood samples, malaria parasite species were identified using nested PCR protocols specific to *P. falciparum* (microsatellite marker Pk2), *P. vivax* (microsatellite marker 3.502), and *Plasmodium malariae* (18S rRNA) as described previously [12]. When there was insufficient DNA for species identification or if no amplification was obtained in this step, samples were reported as “indeterminate species.”

### Statistical Analyses

Statistical analysis was performed with Stata 13.1 and R 3.1.1. Description of the complete distributions of *P. falciparum* and *P. vivax* densities required imputation from the measured densities and their respective distributions of the partition of species among the lower-density parasitemias in which the species were not identified, as well as among the even-lower density parasitemias, which could not be measured. This was possible because the distributions of measured parasite densities were unimodal, with modal values well within the quantifiable range. In this analysis, parasitemia densities of >30 000 000 parasites/mL were excluded because their uPCR estimation was inaccurate, and parasitemia densities of <30/mL were excluded to reduce stochasticity close to the uPCR limit of accurate detection. Estimates of the density function for log_10_ parasitemia densities of *P. falciparum* and *P. vivax* were obtained using the Epanechnikov kernel estimator (Stata command “kdensity”). These were the only parasite species identified in this study, so the probability of infection being due to *P. falciparum* (*P_PF_*; ie, 1 minus the probability of infection being due to *P. vivax*) was calculated from the observed proportion of *P. falciparum* infections, assuming that the species proportions were the same in the lower-density parasitemias for which the species were indeterminate. Imputed data sets (n= 1000) were then created that comprised all of the observed *P. falciparum* and *P. vivax* cases and the indeterminate cases allocated randomly to species, based on *P_PF_*.

Adjustment to accommodate nuclear division in circulating intraerythrocytic asexual *P. vivax* parasites was examined. It was assumed that there is 1 genome/parasite for the first 36 hours of the 48-hour asexual cycle, followed by doubling every 3 hours, until finally there were 16 genomes per parasite in the 3 hours before schizont rupture.

Two scenarios were investigated: completely asynchronous infections (scenario A), in which there is a uniform distribution of parasite ages between 1 and 48 hours following merozoite invasion; and synchronous infections (scenario B), in which parasite ages follow a normal distribution with a mean age µ of 1, 2, 3  …  to 48 hours following merozoite invasion, with a standard deviation of 4 hours. These scenarios require different adjustment factors to convert genomes to numbers of circulating *P. vivax* parasites. For scenario A, the adjustment factor was as follows:

0.809=[36/48+3/(2∗48)+3/(4∗48)+3/(8∗48)+3/(16∗48)].

The adjustment factor for scenario B was as follows:

$$\begin{array}{rl} & \;1/D*\{[\Phi (36,\mu ,4)-\Phi (0,\mu ,4)]+[\Phi (39,\mu ,4)-\Phi (36,\mu ,4)]\\ & \;/2+[\Phi (42,\mu ,4)-\Phi (39,\mu ,4)]/4+[\Phi (45,\mu ,4)\\ & \;\;\;-\Phi (42,\mu ,4)]/8+[\Phi (48,\mu ,4)-\Phi (45,\mu ,4)]/16\}, \end{array}$$

where Φ(*x*, µ,4) is the cumulative normal distribution with mean µ and standard deviation 4 evaluated at value *x*, and D = [Φ (48, µ,4) − Φ (0, µ,4)] is the total area under the density function between 0 and 48 hours (Figure 1). For scenario A, all *P. vivax* parasite genome counts were multiplied by the same adjustment factor to give estimated parasite densities, while for scenario B, each parasite count had a randomly assigned mean parasite age value µ (from the uniformly distributed values 1–48), and that count was multiplied by the adjustment factor corresponding to the value of µ (Figure 1). The adjustments examined represent maximum deviations because they assume that all *P. vivax* parasites in the sample are asexual.

For each data set, the following were performed: (1) analysis with the Kolmogorov–Smirnov test to determine whether the log-transformed data conformed to a truncated normal distribution with parameters (*m* [mean] and *s* [standard deviation]), with the truncations corresponding approximately to the lower and upper limits of accurate quantitation; (2) estimation of the mean *m* and standard deviation *s* of the untruncated distributions of log_10_-transformed parasitemia values, using the maximum likelihood method; and (3) estimation of the tail of the distribution corresponding to the lower densities' truncated segment from the log-normal distribution with parameters (*m; s*).

The first 20 imputed data sets were also used to examine the relationship between parasitemia and study site and patient age. A linear model for log_10_ parasitemia with these covariates and a random effect for participant, to account for multiple measurements. was fitted for each imputed data set. The imputation estimates of the parameter coefficients and their 95% confidence intervals [C]I were calculated [19]. Logistic regression was fitted to estimate relationships between risk of fever and parasite density.

### Ethics Committee Approval

The studies were approved by the Tak Province Community Ethics Advisory Board, the Cambodian National Ethics Committee for Health Research (0029 NECHR; 4 March 2013), and the Oxford Tropical Research Ethics Committee (1015–13; 29 April 2013).

## RESULTS

Of the 7 villages selected for more-detailed studies, 3 (KL, OK, and PDB) are in Cambodia, with 2151 inhabitants surveyed between June 2013 and June 2014. The 4 remaining villages (TOT, TPN, KNH, and HKT) are along the Thailand-Myanmar border, with 2589 inhabitants surveyed between May 2013 and June 2015. Of the population included in the census, 83% participated in the surveys in the Cambodian villages and 67% participated in villages along the Thailand-Myanmar border. The median age of the participants was 21 years; 37% were <15 years old, and 51% were male. The age distributions were similar in the different villages [11].

### Parasite Densities

**Figure 2.**
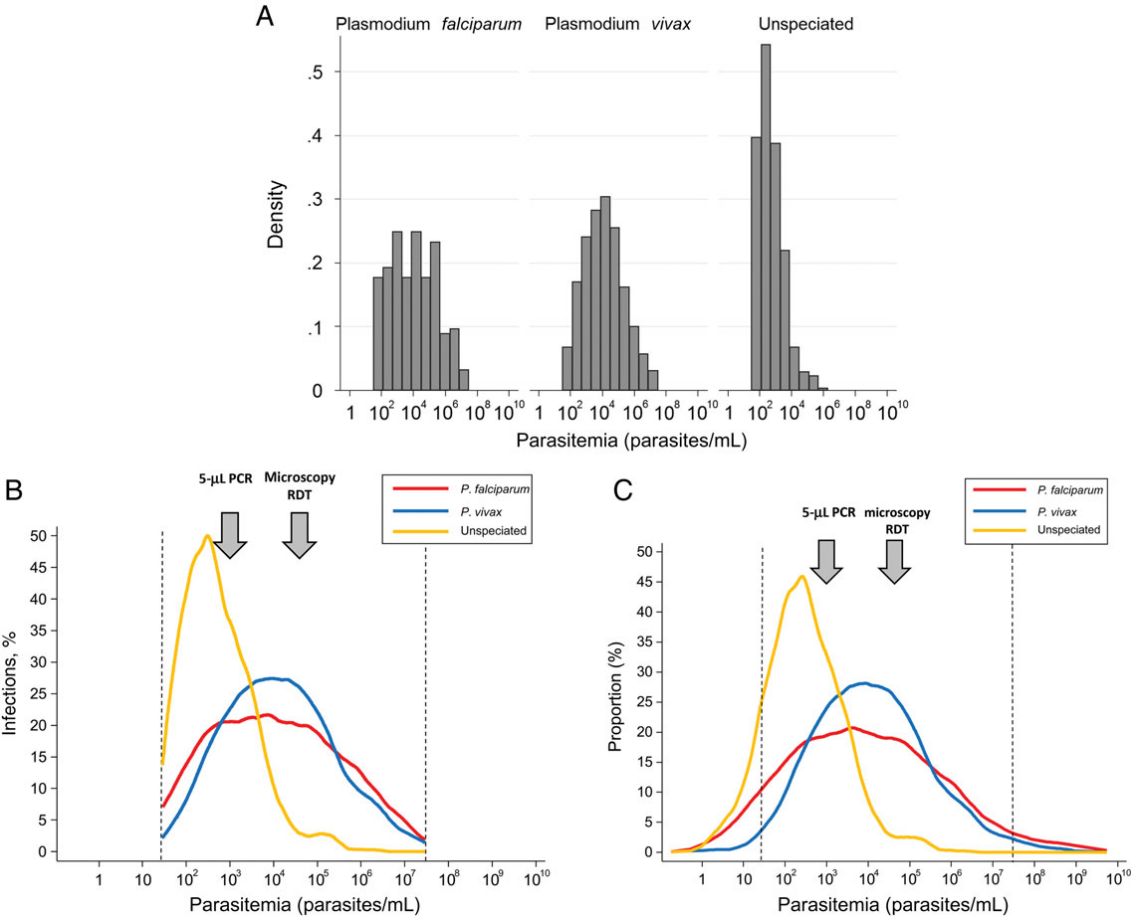
*A*, Histogram showing *Plasmodium vivax* and *Plasmodium falciparum* densities. *B*, The kernel density function based on densities of 30–30 000 000 parasites/mL estimated for each species. *C*, The kernel density function based on all parasite densities estimated for each species. The arrows show approximate lower limits of detection by rapid diagnostic tests (RDTs) and polymerase chain reaction (PCR) analysis of capillary blood specimens (5 µL).

In total, 4740 participants were tested on 16 662 occasions, with a median of 5 tests/participant (range, 1–12 tests/participant). Malaria parasitemia was detected by uPCR testing on 2432 occasions in 1303 participants: 231 infections (9.5%) were *P. falciparum* only, 1553 (63.9%) were *P. vivax* only, 52 (2.1%) were mixed, and 596 (24.5%) had indeterminate parasite species (ie, the specimens were positive for malaria parasites by uPCR, but there was insufficient DNA to determine the species; Figure 2*A*).

Distributions fitted to the quantitated parasite densities resulted in exclusion of 122 infections with a density of <30 parasites/mL and 31 with a density of >30 000 000 parasites/mL (23 *P. falciparum*, 48 *P. vivax*, 3 mixed, and 79 indeterminate infections). The parasite density distributions were log normal and unimodal (see Figure 2*B* and 2*C* for kernel densities). After these exclusions, the uPCR-measured parasitemia levels had geometric means of 9801 parasites/mL (95% confidence interval [CI], 6238–15 399 parasites/mL) for *P. falciparum*, 11 919 parasites/mL (95% CI, 10 311–13 779 parasites/mL) for *P. vivax*, and 476 parasites/mL (95% CI, 406–557 parasites/mL) for the indeterminate species infections.

Overall, *P. falciparum* infections comprised 12.1% of speciated monoinfections (208 of 1713) within the detection limits. Among *P. falciparum* infections, 15.3% had parasite densities of 30–999 parasites/mL, and 11.2% had densities of ≥1000 parasites/mL (*P* = .031). To examine how this affected the estimates of undetected parasitemias, *P_PF_* was estimated by method 1, where *P_PF_* = 0.1214 for all densities, and by method 2, where *P_PF_* = 0.1534 for densities between 30 and 1000 parasites/mL and *P_PF_* = 0.1124 for densities between 1000 and 30 000 000 parasites/mL.

With the uniform *P_PF_* = 0.1214, for the 1000 simulated corrected data sets the mean estimated log density parameters were m = 2.86 (interquartile range [IQR], 2.75–2.97), with standard deviation s = 2.16 (IQR, 2.09–2.22) for *P. falciparum* and m = 3.34 (IQR, 3.33–3.35) and s = 1.60 (IQR, 1.60–1.60) with an asynchronous parasite age distribution for *P. vivax* (scenario A)*.* These estimates were only slightly different for synchronous *P. vivax* parasite age distributions (scenario B): m = 3.25 (IQR, 3.23–3.27) and s = 1.64 (IQR, 1.63–1.66), respectively (Table 1). With the two *P_PF_* values (0.1534 and 0.1124), the estimates changed slightly for *P. falciparum* but not for *P. vivax*; over the 1000 corrected data sets, for scenario A m = 2.64 (IQR, 2.49–2.79) and s = 2.56 (IQR, 2.17–2.33) for *P. falciparum* and m = 3.35 (IQR, 3.35–3.36) and s = 1.59 (IQR, 1.59–1.60) for *P. vivax*, and for scenario B m = 3.26 (IQR, 3.24–3.28) and s = 1.63 (IQR, 1.62–1.65). For both methods, the distributions of the log_10_-transformed parasite densities in all the corrected data sets could be assumed to be normally distributed (*P* > .38 for *P. falciparum* and *P* > .092 for *P. vivax*, both by the Kolmogorov-Smirnov test).

**Table 1.** Estimated Parasite Density Distributions Derived From 1000 Imputed Data Sets

Predicted Log-Normal Distributions	*Plasmodium falciparum*			*Plasmodium vivax*^a^					
				Scenario A			Scenario B		
	Mean	Range	IQR	Mean	Range	IQR	Mean	Range	IQR
*P_PF_* = 0.1214 for all parasite densities (30–30 000 000 parasites/mL)									
Mean	2.86	1.97–3.31	2.75–2.97	3.34	3.31–3.38	3.33–3.35	3.25	3.16–3.33	3.23–3.27
SD	2.16	1.90–2.66	2.09–2.22	1.60	1.57–1.62	1.60–1.60	1.64	1.58–1.71	1.63–1.66
Percentage below LLOQ^b^	25.6	16.3–42.2	23.3–27.8	11.8	11.0–12.6	11.7–12.0	13.6	11.7–15.7	13.2–14.0
Percentage above ULOQ^c^	1.6	1.1–2.1	1.5–1.7	0.48	0.45–0.51	0.48–0.49	0.50	0.42–0.59	0.48–0.52
*P_PF_* = 0.1534 for parasite density of 30–999 parasites/mL; 0.1124 for parasite density 1000–30 000 000 parasites/mL									
Mean	2.64	1.92–3.27	2.49–2.79	3.35	3.31–3.39	3.35–3.36	3.26	3.15–3.36	3.24–3.28
SD	2.26	1.95–2.65	2.17–2.33	1.59	1.57–1.61	1.59–1.60	1.63	1.56–1.72	1.62–1.65
Percentage below LLOQ^b^	29.8	17.9–42.9	26.8–32.7	11.5	10.9–12.4	11.4–11.7	13.3	11.0–16.1	12.9–13.8
Percentage above ULOQ^c^	1.6	1.1–2.2	1.5–1.7	0.48	0.46–0.51	0.48–0.49	0.50	0.42–0.59	0.48–0.52

Abbreviations: IQR, interquartile range; *P_PF_*, probability of infection being due to *P. falciparum*; SD, standard deviation.

^a^ Scenario A describes asynchronous infections. Scenario B describes synchronous infections (SD, 4 hours).

^b^ The lower limit of quantification (LLOQ) was defined as <30 parasites/mL.

^c^ The upper limit of quantification (ULOQ) was defined as >30 000 000 parasites/mL.

**Figure 3.**
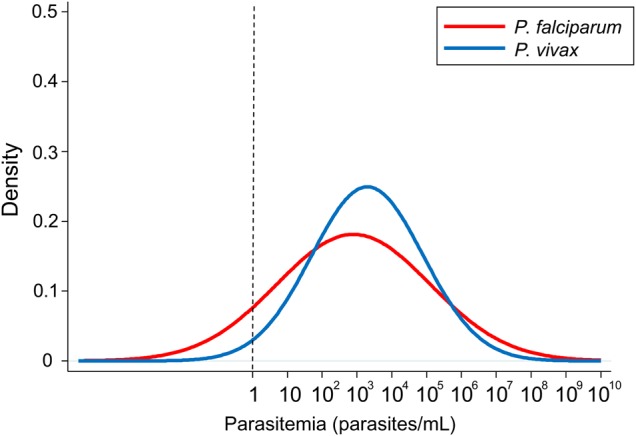
The predicted geometric distributions of *Plasmodium falciparum* and *Plasmodium vivax* densities*.* Results are shown for the mean values (m) and standard deviations (s) of parameters (m,s) of (2.9,2.2) for *P. falciparum* and (3.3,1.6) for *P. vivax*.

**Figure 4.**
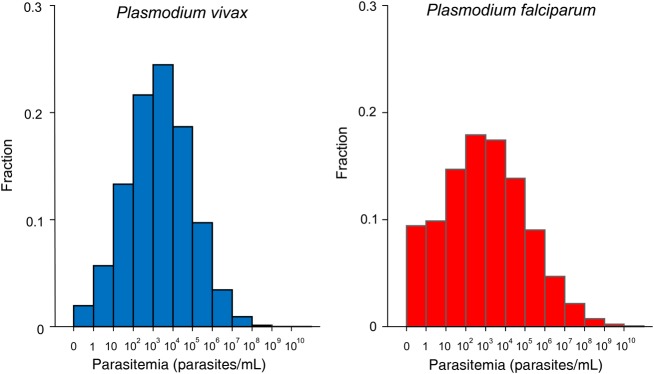
Histogram of the imputed parasite densities for *Plasmodium falciparum* and *Plasmodium vivax.* The height of the bars is scaled so that the sum of the heights equals 1. Results are shown for the mean values (m) and standard deviations (s) of parameters (m,s) of (2.9,2.2) for *P. falciparum* and (3.3,1.6) for *P. vivax*.

After correction for the indeterminate parasitemias, the imputed corrected median (IQR) geometric mean parasite densities for detected infections were 5158 parasites/mL (IQR, 4863–5454 parasites/mL; method 1) and 4627 parasites/mL (IQR, 4382–4940 parasites/mL; method 2) for *P. falciparum* and 4529 parasites/mL (IQR, 4495–4567 parasites/mL) and 4598 parasites/mL (IQR, 4556–4636 parasites/mL) respectively for *P. vivax* with scenario A and 4454 parasites/mL (IQR, 4392 to 4517 parasites/mL) and 4513 parasites/mL (IQR, 4452–4580 parasites/mL) respectively for *P. vivax* with scenario B (Figures 3 and 4).The estimated proportions of undetected infections (ie, those not identified because they were below the limits of accurate quantitation) were 11.8% (IQR, 11.7%–12.0%) and 13.6% (IQR, 13.2%–14.0%) of the *P. vivax* infections for scenarios A and B, respectively, and 25.6% (IQR, 23.3%–27.8%) of those with *P. falciparum.* The proportions truncated at the upper ends of the distributions were 0.5% (IQR, 0.5%–0.5%) for *P. vivax* for both scenarios A and B and 1.6% (IQR, 1.5%–1.7%) for *P. falciparum*. Analysis with different *P_PF_* ratios above and below parasite densities of 1000 parasites/mL had little effect on the estimates, with an estimated 11.5% (IQR, 11.4%–11.7%) or 13.3% (IQR, 12.9%–13.8%) of *P. vivax* infections, respectively, being undetected and 29.8% (IQR, 26.8%–32.7%) of *P. falciparum* infections being undetected. This results in an overall true distribution of asymptomatic infections in these populations as follows: *P. falciparum*, 13%; *P. vivax*, 83%; and mixed, 4%.

### Parasite Densities and Clinical Covariates

**Figure 5.**
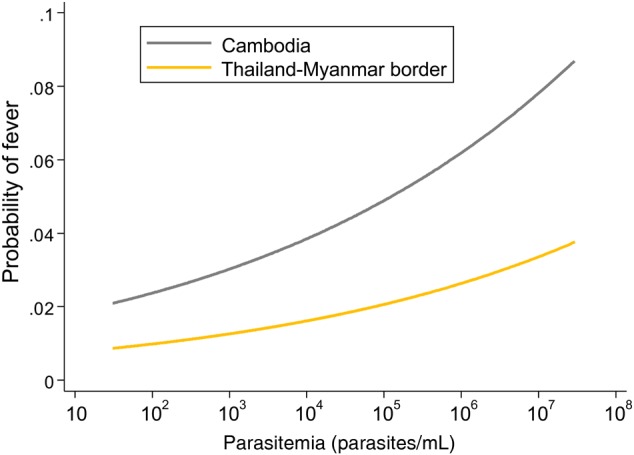
Relationship between parasite density and risk of fever in this study.

Fever (aural temperature, > 37.5°C) was detected on 73 occasions in 68 participants who did not complain of symptoms. Participants in Cambodia had a higher risk of fever than those on the Thailand-Myanmar border (odds ratio [OR], 2.44; 95% CI, 1.43–4.18; *P* = .001). The risk of fever increased with parasitemia density (OR, 1.28; 95% CI, 1.08–1.53 per 10-fold increase; *P* = .005; Figure 5) and was not different between species. Asymptomatic parasite densities were 51.1% lower (95% imputed CI, 10.4%–73.4%) in children, compared with adults, and 31.0% lower (95% imputed CI, 3.7%–50.6%) in Cambodia, compared with the Thai-Myanmar border. Overall, the differences in parasite densities between P. *falciparum* and *P. vivax* malaria were not significant (−10.5%; imputed 95% CI, −41.5% to 36.9)**.**

## DISCUSSION

In malaria-endemic areas of western Cambodia and the Thailand-Myanmar border, >85% of asymptomatic *P. vivax* infections and nearly 75% of asymptomatic *P. falciparum* infections could be detected by uPCR. The distributions of parasite densities were unimodal and log normal, as they are for symptomatic malaria and indeed for many other bloodstream infections. There was no evidence for discrete subpopulations of lower densities. If it is assumed that the malaria species proportions in the lower parasite density range (in which species could not be identified) were consistent with the proportions in the range where species identification was possible, then the geometric mean densities for all detected asymptomatic *P. falciparum* and *P. vivax* infections were similar, at approximately 5 parasites/µL (5000 parasites/mL; 2.5 × 10^7^ parasites/adult).

The uPCR DNA–based measurement can overestimate parasite densities slightly, particularly in *P. vivax* malaria, as parasite genomes in merozoites in developing schizonts and those free in plasma (<0.01% of concomitant intraerythrocytic numbers) and phagocytosed by leukocytes may be quantitated [20]. DNA assessment also does not distinguish asexual from sexual parasite stages, unlike highly sensitive messenger RNA methods [21, 22]. As gametocytes are cleared more slowly than asexual stages from the peripheral blood, particularly in *P. falciparum* infections, a significant proportion of the parasites detected by uPCR in asymptomatic individuals are likely to be gametocytes. In this study, the potential errors related to schizontemia and synchronicity of infection in *P. vivax* infection were examined and found to contribute little to the estimates of geometric mean parasite density or undetected proportions, and measurement errors from free DNA or merozoites were minimized by red cell separation. If the distribution of parasite densities in asymptomatic individuals living in low-transmission settings is visualized as an iceberg, only the top of which is revealed by conventional microscopy or rapid diagnostic tests (RDTs), then high-volume uPCR reveals most of the iceberg and thereby allows prediction of the proportion of individuals who have parasite densities below the level of detection.

These data also suggest that the majority of infected individuals should be detected by widely used methods involving PCR analysis of capillary blood specimens on filter paper, which typically contain only 5–10 µL of blood [23–25]. However, because the limit of detection for these methods is in the parasite density range that contains a large proportion of asymptomatic parasitemias (1–10 parasites/µL), small interassay or intraassay changes or variations in assay performance could have large effects on their malaria prevalence estimates.

Asymptomatic malaria infections in low-transmission settings may be harbored in an individual for long periods in a quasi-steady state [26–33]. If the longitudinal profile of submicroscopic parasite densities mirrors that in the range countable by microscopy, then regular waves of higher densities occur with the sequential emergence of new antigenic variants [27–30, 34]. In *P. vivax* malaria, persistence is enhanced by relapse, which occurs in the majority of infections in Southeast Asia [35–37]. Some of these waves of asexual parasitemia generate transmissible densities of gametocytes [27–30, 33, 34, 38, 39]. Because the prevalence of asymptomatic malaria at any time is orders of magnitude higher than that of symptomatic malaria, the transmission reservoir from asymptomatic malaria is likely to be large. Assuming that a vigorous host response to malaria reduces the probability of onward transmission, particularly in the modern era of antimalarial drug treatment, then, from a parasite perspective, the optimum density to persist at is one that does not generate illness but has the greatest chance over time of generating transmissible gametocyte densities.

Are these parasite density distributions from areas of low, unstable malaria transmission in Southeast Asia relevant to higher-transmission settings, where a much larger proportion of asymptomatic individuals have parasitemias detectable by microscopy? Immunity to blood stage parasites is greater in higher-transmission settings, and splenic function is augmented and, thus, clearance of parasitized erythrocytes is likely to be more rapid, so the net result of higher inoculation rates will be a truncated lower end of the parasite density range, with proportionally fewer densities below the limit of uPCR detection. This is because, with a median sporozoite inoculum of approximately 10 and liberation of approximately 35 000 merozoites from each hepatic schizont, the median blood parasite density following hepatic schizogony of each inoculated brood in an adult (blood volume, approximately 5000 mL) approximates 70 parasites/mL (which is above the current level of uPCR detection). The greater proportion of very low-density *P. falciparum* infections, compared with *P. vivax* infections, in this study may reflect the currently lower transmission of this species in the study areas and, thus, the greater probability that an individual was being sampled while their infection was being eliminated, whereas *P. vivax* infections were acquired both from inoculation and more-frequent relapse [35]. In contrast, in high-transmission settings, the upper range of densities of asymptomatic infections is known to be extended as premunition elevates the pyrogenic density [27]. In such high-transmission areas, a higher proportion of the population is parasitemic on the basis of microscopy or RDT findings, and asymptomatic parasite densities often extend up to 10 000 parasites/µL. Age is a greater determinant of parasite density distributions than in lower-transmission settings. A recent cross-sectional survey conducted in an area of previously high transmission in Papua New Guinea two years after distribution of insecticide-treated bed nets used sensitive nucleic acid based methods of parasite detection and estimated geometric mean parasite densities of 42 genomes/µL for *P. falciparum* and 8 genomes/µL for *P. vivax* in identified infections [21]. However, densities were substantially higher in young children (for *P. falciparum*, approximately 650 genomes/µL; for *P. vivax*, approximately 140 genomes/µL). Overall, it is likely that more of the “iceberg” of the parasite density distribution in asymptomatic people will be detectable in the microscopy or RDT detectable ranges in settings of high transmission, compared with settings of low transmission.

These data suggest that uPCR assessment of parasite densities in population cross-sectional surveys provides accurate characterization of the true prevalence of malaria in the community in all transmission settings. If the density distributions reported here are general properties of malaria, then the regional and total global burden of malaria parasites can be calculated, and these data used to estimate accurate selection pressures provided by drugs and other interventions.
